# Gait Recognition as an Authentication Method for Mobile Devices

**DOI:** 10.3390/s20154110

**Published:** 2020-07-23

**Authors:** Matei-Sorin Axente, Ciprian Dobre, Radu-Ioan Ciobanu, Raluca Purnichescu-Purtan

**Affiliations:** 1 Faculty of Automatic Control and Computers, University Politehnica of Bucharest, RO-060042 Bucharest, Romania; matei_sorin.axente@stud.acs.upb.ro (M.-S.A.); ciprian.dobre@cs.pub.ro (C.D.); 2 National Institute for Research and Development in Informatics, RO-011455 Bucharest, Romania; 3 Department of Mathematical Methods and Models, University Politehnica of Bucharest, RO-060042 Bucharest, Romania; raluca.purtan@mathem.pub.ro

**Keywords:** mobile devices, smartphones, authentication, privacy, gait detection

## Abstract

With the rate at which smartphones are currently evolving, more and more of human life will be contained in these devices. At a time when data privacy is extremely important, it is crucial to protect one’s mobile device. In this paper, we propose a new non-intrusive gait recognition based mechanism that can enhance the security of smartphones by rapidly identifying users with a high degree of confidence and securing sensitive data in case of an attack, with a focus on a potential architecture for such an algorithm for the Android environment. The motion sensors on an Android device are used to create a statistical model of a user’s gait, which is later used for identification. Through experimental testing, we prove the capability of our proposed solution by correctly classifying individuals with an accuracy upwards of 90% when tested on data recorded during multiple activities. The experiments, conducted on a low sampling rate and at short time intervals, show the benefits of our solution and highlight the feasibility of an efficient gait recognition mechanism on modern smartphones.

## 1. Introduction

Modern smartphones usually employ one or a combination of the following methods to validate the identity of the user: PIN, password, pattern, fingerprint sensor, or facial recognition. These five mechanisms can be grouped into two categories based on which factor they represent in the three-factor authentication model (https://blog.gemalto.com/security/2011/09/05/three-factor-authentication-something-you-know-something-you-have-something-you-are/). The first three methods are based on something known and are usually created by the user. The main issue of these approaches is the human factor, the education of the user regarding cybersecurity being decisive in the strength of the password. Furthermore, the large number of passwords a user has to remember leads to duplicate and low-strength passwords. Using something owned by the user may enhance these methods, but implies using an additional software product (e.g., soft token) or physical device (e.g., hard token). Fingerprint scanning and facial recognition belong to the field of biometrics and represent some characteristics of the user. Although at first impression they may seem harder to crack, these methods are weak against presentation attacks, and the technology needed to create such an attack is evolving (e.g., fingerprint extraction from regular photos).

In order to stay ahead of malicious entities, the virtual environment is in need of new authentication mechanisms that can enhance the preservation of private data and that rely less on the active contribution of the user, thus ensuring a strong defence and reducing the chance of human error. Thus, the objective of this paper is to develop a new security mechanism that may be easily integrated into a multiple factor authentication model, by triggering existing authentication methods when the current user is not identified as the expected person. To begin with, the method should be able to determine the identity of a previously stored user or an attacker with a high level of confidence. In addition, the whole process should be completed within a relatively short time interval (both for data collection and processing), in order to provide a seamless experience. In terms of resources, the size of a sample should be small since it will only contain values from the sensors, while the samples need to be stored locally in order for the mechanism to work without depending on a network connection. Finally, all the sensors that are used should be available on the majority of Android smartphones and the resulting application should work on most of the Android versions.

The proposed method is therefore based on collecting data from the sensors available on Android smartphones while the user is walking. The information gathered this way is then used to make a statistical analysis of the gait (manner of walking) of the user [1], which is then stored as a histogram on the device. To evaluate the efficiency and correctness of this method in real-life scenarios, it is integrated in an Android application which runs on a modern smartphone carried in the trouser pocket. This paper discusses the validity of this method for other activities than walking and its applicability to other sensors, through the use of a data set consisting of data collected from users engaged in different activities, experimental data collected through the implemented application.

Through experimental evaluation, we are able to show that the method proposed in this paper achieves an accuracy of upwards of 90% on walking samples from a standardized data set through the application of histogram similarity on accelerometer data. The addition of the gyroscope sensor in the application enhances the algorithm and successfully classifies all the test samples of gait data in three different scenarios: walking on a flat and inclined treadmill at 4 km/h, and also at a speed of 8 km/h. We also test our solution on other activities and show that a high rate of success can be achieved even then. The tests also reveal that the proposed solution is an efficient method of identifying users and can be extended to a continuous authentication algorithm that can easily run on smartphones without rapidly draining the battery.

The rest of this paper is structured as follows: Section 2 discusses the research in the domain of gait recognition and similar solutions, whereas Section 3 presents the motivation for our work, while also describing the proposed solution and its implementation in Android. Section 4 consists of a statistical evaluation of the algorithm in multiple scenarios, showing how its performance is influenced by the values of its main parameters. Finally, Section 5 presents our conclusions and future work.

## 2. Related Work

Studies regarding gait recognition have been conducted for decades, being initially focused on the ability of people to recognize themselves or others by their walking mannerism. There are two main directions in this domain. The first one is represented by the analysis of video samples using image processing techniques. For example, one solution proposes a new approach in automatic gait recognition which relies on the symmetry of walking cycles [2]. Image filters (grayscale and the Sobel operator for edge detection) are applied to the frames of the video to extract the silhouette of the person and then the outline. Symmetry is computed based on the midpoints of the sectors defined by the points on the outline. Another visual approach is the use of computer vision. An example of such a classifier explores the possibility of identifying gait by constructing both a motion model and a structural model of the thigh [3]. The model is similar to a pendulum, with the hip acting as a pivot and the knee acting as the weight. The results show that a perfect classification is possible with the proposed solution.

The visual approach is suitable for identifying individuals at a distance, without contact with the subject. This means that these mechanisms are suitable for surveillance systems but cannot be used at close range for authenticating a user on a smartphone. The second direction of research is more appropriate for identity validation in a close-range scenario. This direction relies on data collected from motion sensors to determine the gait model of a person and is more suitable for smartphone integration since multiple types of motion sensors are embedded in modern mobile devices.

Motion sensors offer a lot of information about the state of the phone and how it is being used, while their variety allows for a multitude of approaches. For example, the accelerometer can be used to record data while triggering the vibration in certain patterns as a means of creating a device fingerprint in a network [4]. A great example of how motion sensors can enhance classical authentication methods is presented in [4], where the authors propose gathering data from the touch sensors embedded in the screen, the accelerometer, and the gyroscope to calculate the geometry of the hand of the user. Not only can the process of typing a password be enhanced by this mechanism to transform it into a two-component authentication scheme, it is itself a multi-factor model, since an attacker has to replicate three different types of data to gain access.

The two aforementioned methods are enhancements to existing authentication solutions and require an action from the user. An advantage of gait recognition is the passive non-intrusive nature of this mechanism which makes it more convenient than password authentication, since it does not rely on something that the user knows. This also means that such a mechanism can be used as a form of continuous authentication because collecting data from the motion sensors on a smartphone is not a complex task, as exemplified by mobile games that have been using this information for quite some time (for example, steering in driving games).

Identification using accelerometers is a relatively new direction in gait research. Most of the studies use a MEMS (Micro-Electro-Mechanical-System) device to record acceleration data, and the algorithms are focused on creating a statistical model of a cycle, walking being considered symmetric. The analysis is mainly done in two ways: cycle length or histogram comparison. Cycle length can be determined through multiple methods usually using the vertical axis, for example by extracting local minimums and maximums [5] or by calculating the mean value of acceleration and subtracting it from the signal to identify the zeros or observation points [1]. The second method uses histograms to compute a statistical method of the gait. In [1], the two methods were compared on the same data set, resulting in an EER (equal error rate) of 9% for cycle length and a better 5% EER for histogram similarity.

As mentioned earlier, the cyclic nature of gait is one of the main focuses in literature. In [6], a method of identifying the cycles of gait is presented and evaluated. One disadvantage of the Android platform in particular is mentioned: the sampling rate passed to a sensor listener guarantees that a sensor event will be received until the end of the sampling interval, but not that the intervals are equal. However, the frequencies supported by the sensors embedded in current smartphones increased with time, which means the interpolation of data may not be necessary anymore. The proposed cycle detection algorithm relies on salience vectors, which can be easily computed, thus suitable for the mobile environment. The vectors are used first to estimate the length of a cycle and then to detect it. Before comparing the new sample with the database, pre-processing is done to remove unusual cycles and identify the best candidate. Comparison is done through Majority voting and Cyclic Rotation Metric. Based on a data set consisting of walking on a straight surface but also up and down stairs, MV achieved an EER of 28% and CRM 21.7%. However, in terms of time, the mean time of computing MV was 5685 ms and 1864 ms for CRM. These times may have changed drastically with the evolution of mobile devices.

Motion sensor-based gait recognition is similar in accuracy to computer vision gait analysis, but more environmental factors should be taken into consideration for the latter. The required video cameras can be sensitive to lighting conditions and may misidentify a subject in case of a significant difference in clothing. Attire can influence sensor data as well (for example, if the smartphone is kept in a pocket), but it is easier to relocate the device in a more appropriate place or to enrol another data set. Other factors that may alter gait data are: abnormal gait caused by injuries or chronic affections, age, additional weight carried by the test subject, conscious control, the terrain or its inclination, footwear, and fatigue.

According to [7], the structure of current gait recognition methods based on MEMS can be split into three parts: sensor data being collected using the devised Micro-Electro-Mechanical-System, the computation of the statistical model, and finally the comparison between the newly acquired data and the patterns already enrolled in the algorithm. Although most of the studies use specially configured circuit boards with accelerometers for the three axes, successful experiments have been reported that use commercially available smartphones, especially since modern mobile devices have sensors that are easily configurable through the API in the operating system. The most common frequencies used for data collection are between 50 and 100 Hz, but go as low as 25 Hz. Combined signals are preferred to raw data since the orientation of the device does not affect the resulting gait signal. The authors also show that there is a constant growth in the number of gait-related research, which can be explained by the variety of real-life applications that such mechanisms could have. These include (but are not limited to) cybersecurity, surveillance, detection of disorders of the locomotor system, and fitness.

In a more recent survey [8], four steps are identified: data acquisition, data pre-processing, walk detection, and analysis. The first step is handled by the mobile device, but we also consider the possibility of handing the third step to the device, through the use of the Activity Recognition API provided by Google and cycle detection using the step detection virtual sensor. This would help reduce the impact of the algorithm on the resources of the mobile system. As mentioned, the two approaches in regard to gait recognition are sensor based and video based. The survey provides a comparison between implementations using one or both methods. Computation wise, statistical methods, and machine learning are generally used. The values for accuracy are usually above 90% with EER values ranging from 3% up to 20–30%. However, a common issue with the evaluation of the algorithms in this category is the relatively small number of test subjects and the movement being recorded in an environment which tends to be idealistic in one or more aspects.

## 3. Gait Detection as an Authentication Mechanism

This section presents a solution for using gait detection as a security mechanism for Android devices, starting with the motivation of this research, the proposed algorithm, and its implementation in Android.

### 3.1. Motivation

As mentioned earlier, most of the modern smartphones offer a few methods of authentication. The most common smartphone authentication scheme is the password, being used not only by mobile devices but also by the majority of websites and apps in combination with an account. However, its main weaknesses are actually consequences of this popularity. The password is usually chosen by the user and so, in order to be effective, it heavily relies on the education of its creator regarding cybersecurity. It is thus the duty of the user to create a complex and unique password. LastPass, a business focused on password management, conducted a study in 2017 (https://www.theseus.fi/bitstream/handle/10024/151533/Evaluating%20Password%20Managers%20for%20Enterprises.pdf) which revealed that the average number of passwords an employee manages is 27, a value that can only increase with time, due to more websites and applications requiring authentication to provide access to their content or personalize the experience. Focusing only on the business sector, this number increases to 191 according to the same survey. Thus, it is understandable why a person would be inclined to either use passwords that are short and easy to type and remember, or to simply reuse a small number of passwords.

The PIN can be considered a particular case of password, only it is limited to four digits, which greatly reduces the number of possible combinations and makes it extremely vulnerable to shoulder surfing, since the time required to see another person authenticate using this method is rather short. Furthermore, the layout of the numerical keyboard on a smartphone may be easy to reproduce for a trained eye. The same applies to the pattern lock with the addition of smudge attacks. This method is based trying to recreate a previously entered pattern by tracing the oily residue left by the finger on the screen. A 2010 study [9] analysed the feasibility of such an attack on the Android pattern lock, which consists of a three-by-three grid of dots that allows 389,112 different combinations, taking into consideration the restrictions (for example, if a point is directly on the line between two other selected points, it will be selected as well). The study found that the smudges are actually remarkably persistent, the authors managing to partially recreate the pattern in 92% of the test cases and to completely identify it in 68% of them by filming the screen of a device with different camera and lighting setups. Even in the worst scenarios they managed partial recovery in 37% of the worst setups and full recovery in 14% of them. Similar to the password authentication, the length and complexity of the pattern is directly proportional to the effort required to reproduce it.

Another approach to authentication is biometry, such as fingerprint scanning and facial recognition. The statistical analysis of biological characteristics and the possibility of their use as a means of authentication is being researched on a range of human physical characteristics. Although the initial impression is that these methods are better in terms of identifying a fraudulent authentication attempt, they have some weaknesses. Fingerprint identification is based on the analysis of the particularities of the fingerprint. Both optical and thermal fingerprinting techniques are vulnerable to fake fingerprints created from moulds or reconstructed from images [10], including on Android devices [11]. Facial recognition techniques are similar to the minutiae extraction, only instead of ridges they use the shapes, positions and dimensions of the eyes, jaw, nose, and other distinctive features of the face, but suffer from similar problems (https://www.wired.com/story/hackers-say-broke-face-id-security/).

Thus, as shown in this section, each smartphone authentication scheme is flawed in some way [12], so the development of novel mechanisms to validate the identity of a user has an important role in patching the weaknesses of existing methods by using them in a multi-factor model. In the next section, we propose a mechanism that uses gait detection as an authentication mechanism on mobile devices.

### 3.2. Proposed Solution

We propose a long-running service triggered periodically to record motion data during walks, used to validate the identity of the user. We thus aim to devise a mechanism which can secure sensitive data on the device if it is stolen or lost during on-foot travel. Since this method is not in real time, it cannot be employed for on-the-spot authentication.

The proposed solution for identification through gait detection assumes that a mobile device collects data from its user through the various available motion sensors. According to the official documentation (https://developer.android.com/guide/topics/sensors/sensors_motion), an Android device can have the following sensors:

- accelerometer—measures acceleration along three axes including gravity
- uncalibrated accelerometer—similar to the accelerometer without bias compensation
- gyroscope—measures the rotation rate as angular speed along three axes
- uncalibrated gyroscope—similar to the gyroscope without drift compensation
- gravity—measures the force of gravity decomposing it in vector components along the three axes
- linear acceleration—measures acceleration along three axes excluding gravity
- rotation vector—measures the vector components of the rotation along the three axes
- significant motion—triggered when a movement is detected and then deactivates, and it does not measure the properties of the detected event
- step counter—measures the number of steps taken since the last reboot of the sensor
- step detection—detects steps and it is used to trigger the step counter sensor.

In this article, we focus on the accelerometer and the gyroscope, which are two sensors that are present in most Android devices, including older models, which increases the cover area of our proposed solution. Furthermore, they are always hardware-based, which increases the data accuracy and ensures that it does not depend on other factors. Both sensors offer three values per sample, for each of the three axes.

A common hardware component for the acquisition of motion data on smartphones is the BMI160 inertial measurement unit fabricated by Bosch, which contains both an analog accelerometer and an analog gyroscope. It is a tiny component, measuring 3 mm in length, 2 mm in width, and 0.83 mm in height. It is a perfect example of how complex the sensors on modern smartphones have become. It detects 9-axis motion and has an advanced power management system. The two sensors have three different operating modes: normal, suspend, and low power operating mode, which means that the sensors are able to quickly switch from normal mode to suspend and backwards, thus optimizing the power consumption. This unit consumes from 925 μA in full operation mode for both sensors as low as 3 μA in suspend mode. It also features a wide power supply voltage range from 1.71 V to 3.6 V. In terms of data rate, the accelerometer supports from 12.5 Hz to 1600 Hz and the gyroscope from 25 Hz to 3200 Hz in normal power mode. Clearly, these sensors are more than capable of handling a frequency that is suitable for the proposed algorithm without delaying other processes.

Thus, data from these two sensors are being collected and stored in the background while the user explicitly allows this. Furthermore, data from the last interval (which can be configured by the user in the settings menu) are always stored and continually updated even if data collection is turned off. When the user wants to log into their smartphone, the most recently collected data are used as an input into our proposed algorithm, which compares them to the already collected data and decides whether the user attempting to log in is the actual owner of the device. In order to compare collected data, we compute histograms for each sensor output and apply histogram intersection [13,14] in order to compare histograms. Although histogram intersection is used particularly in image recognition, we believe that it can be suitable for gait recognition since the acceleration and rotation on a certain axis are suitable identifiers for a user’s behaviour. In preliminary testing, the exclusive use of the gyroscope proved to have poor performance in the classification of the samples; however, when paired with the acceleration data, the accuracy increased.

The histogram intersection algorithm computes the similarity between an input histogram $H_{1}$ and a collected $H_{2}$ as:

(1) $$similarity=\sum_{i=1}^{n}\frac{min(H_{1i},H_{2i})}{\sum_{i=1}^{n}H_{2i}}$$

As seen above, the minimum of the two histograms is computed by selecting the lower value of a bin from the histograms. This is then compared to the histogram from the database based on the differences in the values of the bins, the aim being to minimize these differences, thus maximizing the correlation.

Although gait recognition studies show a tendency towards using a combined signal that unifies the three measured signals from a sensor (i.e., from the three axes), as exemplified in [1], for our solution, we extend this for all three vector components of the acceleration and gyration. The idea is based on the technique used to classify images based on colour indexing as explained in [13]. Creating histograms from three-dimensional data are not only a possibility but can be developed into a high accuracy classifier. Therefore, assuming that $x_{i}$, $y_{i}$ and $z_{i}$ are the three measured signals of a sensor with index *i*, the value $X_{i}$ saved in the histogram for the OX axis is computed as shown below (and similarly for the other two axes):

(2) $$X_{i}=1+arcsin(\frac{x_{i}}{\sqrt{x_{i}^{2}+y_{i}^{2}+z_{i}^{2}}}),i=\bar{1,n}$$

In Equation (2), the inverse trigonometric function smoothens the result and reduces the domain to values between –1 and 1. In order to compute the similarity correctly (as shown in Equation (1)), we need to add 1 to the result so that the computed values are positive and can be easily normalized.

The present paper focuses on the potential architecture of a continuous gait recognition mechanism which runs in the background on an Android smartphone. The main focus of smartphone vendors is battery life. This is made clear by the increasingly aggressive restrictions on background processes introduced with each new version. To evaluate the feasibility of such a mechanism, we must first validate that it can run on the target device. Our intention is to develop the experimental architecture with simple yet effective computations, in order to ensure uninterrupted processing. However, the aspect of integrating and testing different gait recognition algorithms was considered and the architecture is modular from this perspective, facilitating the swap of the user identification algorithm. Histogram comparison was chosen as a starting point due to the reduced computational effort, in order to test the limits of background processing in Android and also reduce battery usage.

### 3.3. Implementation

The aim of this research is to develop an Android application which allows the user to trigger data recording services to perform the analysis of gait that combines the regular Android-specific locking methods (as shown in Section 3.1) with gait-recognition in a multi-factor mechanism. We believe that a great advantage of gait recognition is that it does not require active cooperation from the user, which means that the experience of using such an algorithm would not become disruptive if employed properly. Thus, a continuous authentication mechanism that would secure the information stored on a mobile device in case of theft may be a feasible solution to enhance the security of the sensitive data that a smartphone holds.

Thus, we implemented an Android application consisting of two main components. Firstly, there is a single activity which exchanges a number of fragments which serve different functions: informing the user of the hardware sensors available for the device, showing the live reading from selected sensors and also illustrating this data in a chart for better visualization. However, the main function of this application is to configure and trigger data recording services for enrolment and validation. The algorithm used to perform motion sensor readings can be run from the app as well, to allow the users to familiarize themselves with the process. However, this method is only meant to exhibit the behaviour, since it can be easily interrupted by any other application which takes the foreground (such as a phone call) or by the sleep timer of the display, which can be overridden using Android system flags, but impacts battery life.

To avoid these issues, we employ a second component based on foreground services (https://developer.android.com/guide/components/services). These Android components are able to run in background continuously by being attached to ongoing (non-dismissible) notifications, the foreground of the service. The motivation behind this choice is mainly based on the background restriction by the operating system which grow more aggressive with each update in order to preserve battery life. Among the main focuses of the changes introduced by Android 9 (https://developer.android.com/about/versions/pie/android-9.0-changes-all) is the prohibition of sensor access to all applications and services running in background henceforth, leaving foreground services as the only solution for uninterrupted motion data collection.

**Figure 1:**
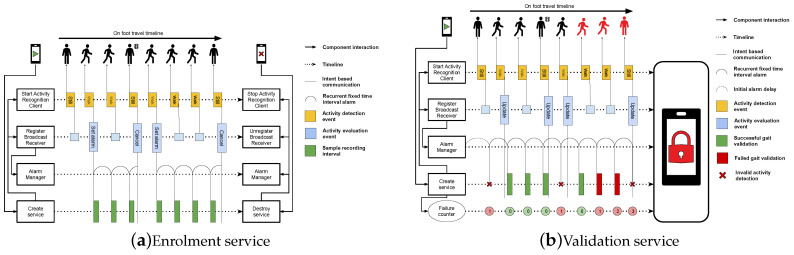
Interaction of components within the gait recording services.

In addition to system aspects, the activity of the user must be taken into account. There is no use in collecting data when the device is still, or in other activities that are not meant to be processed by the algorithm. In addition, walking may be interrupted at any point of the recording, so samples which contain a change of activity need to be discarded. In order to implement this mechanism, we employ activity recognition services provided by Google. There are two available options, the Activity Recognition Sampling API and the Activity Recognition Transition API (https://developers.google.com/android/reference/com/google/android/gms/location/ActivityRecognitionClient). Both implementations use burst of information from low power motion sensors to determine the activity. For the experimental build, we chose the Sampling API, which trades battery consumption for increased control over the detection interval and additional information regarding possible activities, beyond the most probable one with confidence levels. The transition API uses additional processing to discard false positives, but it was difficult to observe transition events while testing. In a production application, the transition API seems to be the better option, since it is slightly more accurate and more efficient in terms of power usage. Currently supported activities are: still, tilting, on foot with walking and running as sub categories, on bicycle and in vehicle, with more values to be added in the future. The role of the Activity Recognition Client is to inform the algorithm when to trigger or cancel data recording, in order to ensure that the sensor information is relevant for the histogram comparison. Inside the foreground services, activity recognition serves two purposes as shown in Figure 1. From the perspective of the enrolment, activity recognition informs the service when information relevant to gait processing can be recorded. From the perspective of validation, the detection only updates the value for the current activity. If this activity can be processed by the algorithm, data recording is started and the resulting data is passed to the histogram comparison algorithm. Otherwise, the validation attempt is considered failed and a failure counter is incremented. This counter is meant to account for potential stops during on foot travel, such as crossing an intersection or using a mean of public transportation, and can be set by the user based on their knowledge of the route. In both cases, the activity detection events are sent as Android intents to the registered broadcast receiver. This component is responsible for determining the validity of the activity in terms of processing, in order to set and cancel the recurring alarm or start and pause the foreground services. Additionally, if an activity event which cannot be processed is received while recording is in progress, the samples in that cycle will be discarded, depending on the activity detection interval and data recording duration.

To avoid draining the battery by interrogating the sensors continuously, the services should be started periodically. The recommended solution in the Android environment is the work manager which uses a combination of implementations available in different versions of the operating system. We experimented with using periodic work requests but discovered a minimum interval between subsequent calls of at least 15 min, which is too long for our use case. In the end, we used a repeating alarm which sends intentions to a service approximately every minute. This interval can be configured by the user, but one minute is the shortest interval which can be targeted in newer Android versions.

We define two services. The enrolment service is meant to create or add to a personal set of samples which the user ensures is their gait. When the user taps the corresponding button, the service is started. During its creation, it registers for activity recognition events through a broadcast receiver. When the user transitions to a valid activity (walking) for recording, the repeating alarm is triggered and the configured number of samples are recorded every minute (this can also be configured by the user). When an invalid activity is detected, the alarm is cancelled and, if a sample was being recorded during the transition, it is discarded. An example of the functioning of the enrolment service is described in Figure 1a. The user of the device is walking, with a pause at a pedestrian crossing.

The validation service also registers for activity events upon creation, but it is triggered independently of the current activity. When the user taps the corresponding button, the repeating alarm is set, and the activity detection broadcast receiver is registered. When the service starts, it checks the current activity. If it is an invalid activity, a counter for failed attempts is incremented; otherwise, a number of samples (configured by the user) is recorded and compared to the database. If the samples fail the validation, the failed attempts counter is increased. The proposed approach is to allow the user to configure a limit of failed attempts, after which the app will require a password to be unlocked. The intention is to allow the user to run the validation service taking into account any stops (for example, waiting to cross an intersection) or using public transport. The functioning of the validation service is described in Figure 1b. In this example, the user is walking and stops at a pedestrian crossing. Shortly after, the mobile device is stolen and the attacker continues walking to evade the victim and then stops. Three validation attempts are triggered after the device is stolen, two recordings which determine the attacker is not the expected user and one invalid activity, which trigger the mechanism meant to secure sensitive data. Additionally, the first failed attempt could trigger a secondary authentication method (separate from the default ones of the device and from the one used if the device is recovered after the failure limit was reached), which can be turned off at the next successful validation, within the failure limit.

For the experimental version of the application, the activity detection sampling interval was set to 0, corresponding to “as soon as possible”, with each event showing a notification with the type of the activity and a timestamp, in order to provide more clarity on the performance of the client. The data recording interval was set to one minute, which is the lowest value which is guaranteed by the operating system, as previously stated. For the other parameters, the proposed values are: five seconds of recording per data sample, a sample rate of at least 100 Hz, three samples per validation attempt (with the result being the result of the majority) and a number of two to three allowed consecutive validation failures, depending on the length of the anticipated stops.

From the perspective of storage, for the experimental application, we integrated the Firebase Firestore service to allow us to collect data from testing subjects in a centralized database. This implies that the validation service will retrieve the necessary samples from the database right after its creation. For an offline approach, we propose to store the samples locally and encrypt them, possibly using other biometric data as key. This key can be acquired through the biometric prompt, a recent improvement in the Android SDK which enables developers to easily integrate fingerprinting, facial or iris recognition (depending on device capabilities) in their applications.

In terms of processing, the data for the accelerometer and gyroscope sensors were recorded simultaneously following the same values for duration and sample rate. The histograms are computed using the same number of bins since the number of sensor events is equal. To generate the comparison score between two samples, we use the average of the values obtained by comparing the acceleration and the rotation histograms, respectively.

We initially used a Python implementation to take advantage of the highly optimized methods available in the NumPy library for histogram creation and comparison. For integrating Python into Android, we employed QPython (http://www.qpython.com), which is a Python interpreter designed for Android that offers multiple libraries which can be easily integrated. With the addition of AIPY (http://www.aipy.org) (which offers mathematical libraries for Python in Android), we were able to implement the solution presented in Section 3.2.

However, this approach was not native to Android and raised compatibility problems during integration. Thus, an equivalent implementation was done in Kotlin, which is the most popular development language on the platform besides Java. Another reason for this choice is Kotlin Native, an emerging technology which enables the code to be run on multiple platforms through native binaries. By separating the computation from the platform specific code, a library able to also run on iOS devices can be developed. In order to manage background processing both for the application and the services, we use ReactiveX libraries, RxJava (https://github.com/ReactiveX/RxJava) with the RxKotlin (https://github.com/ReactiveX/RxKotlin) extensions and RxAndroid (https://github.com/ReactiveX/RxAndroid) schedulers, following the pattern described in this Kotlin Academy article (https://www.kotlindevelopment.com/reactive-sensor-monitoring/). Android was chosen as a personal preference, the two main reasons being experience and the open-source nature of this operating system.

To illustrate the potential use of the algorithm in a multi factor authentication scheme, the Firebase Authentication service was integrated. The proposed behaviour is to perform a logout and prompt the user to login through other means upon application start, if the identity could not be confirmed between uses. If the mechanism is integrated in a launcher, the device will be locked and require authentication through pin, password, or biometrics at the next unlock.

## 4. Evaluation

This section presents the evaluation of the gait detection solution proposed in Section 3.

### 4.1. Experimental Setup

For the development process, a Lenovo K6 smartphone was used. This model was released in November 2016; it has a 1.4-GHz octa-core processor, 2 Gb of RAM, a five-inch LCD capacitive touchscreen and it came with Android 6.0 Marshmallow. It also comes equipped with the BMI160 inertial measurement unit fabricated by Bosch described in Section 3.2. Later, the development was continued using a Huawei P20 lite model running Android 9.0. First, released in 2018, the P20 range helped the growth in popularity of the Huawei brand, which makes it a great candidate for initial validation, being a quite common device amongst Android users. The model features a HiSilicon Kirin 659 chipset, an octa-core (4 × 2.36 GHz Cortex-A53 and 4 × 1.7 GHz Cortex-A53) processor, 4 GB of RAM memory, and 32 GB of storage capacity.

For testing the accuracy of our proposed solution, we also used the “Daily and Sports Activities Data Set” from the UCI Machine Learning Repository (https://archive.ics.uci.edu/ml/datasets/daily+and+sports+activities), since it offers more varied information than we were able to collect with our application, on a more varied array of people. The set contains 19 different activities, each containing data recorded from eight different subjects, with sixty samples for each subject. The readings were done at a sampling rate of 25 Hz, which is quite low but suitable for testing the limits of our proposed algorithm. The low frequency is also good in terms of resource usage, since a background service reading sensor data at a high frequency can quickly drain the battery, so it is important to develop a mechanism that ensures a high enough accuracy with a low enough sampling rate. This is the compromise needed to successfully apply this algorithm on regular smartphones. A sample in the data set has information for five different placements of the recording device: torso, left arm, right arm, left leg, and right leg. For each placement, the data for three sensors (accelerometer, gyroscope, magnetometer) is recorded along the three axes. A sample stores data for 5 s of readings at a rate of 25 Hz. When using the samples for evaluating our solution, 50 out of the 60 samples recorded per subject were used as input data, while the remaining 10 samples were used for testing.

### 4.2. Results

For the first step in our evaluation, we focused on classifying the users by finding the sample in the database which maximizes the score computed through histogram comparison. The testing sample was evaluated against each sample in the enrolment set regardless of user. We looked at the results collected in the “Daily and Sports Activities Data Set” when the sensors were placed on the right leg, and focused only on the accelerometer, which is a sensor found on most Android devices. Based on the histogram similarity solution proposed in Section 3, we measured the user and activity detection accuracy for the 19 activities in the data set: sitting, standing, lying on the back or on the right side, ascending or descending stairs, standing or moving in an elevator, walking on concrete, walking on a treadmill (with 4 km/h, 8 km/h or on an incline), walking on a stepper, cross training, horizontal or vertical cycling, rowing, jumping, or playing basketball. Although gait is defined as a person’s manner of walking, we tested our algorithm on a multitude of activities because it is important for our Android application to be able to differentiate between various activities, and only collect and analyse data relevant to walking or running, and not to other actions.

**Figure 2:**
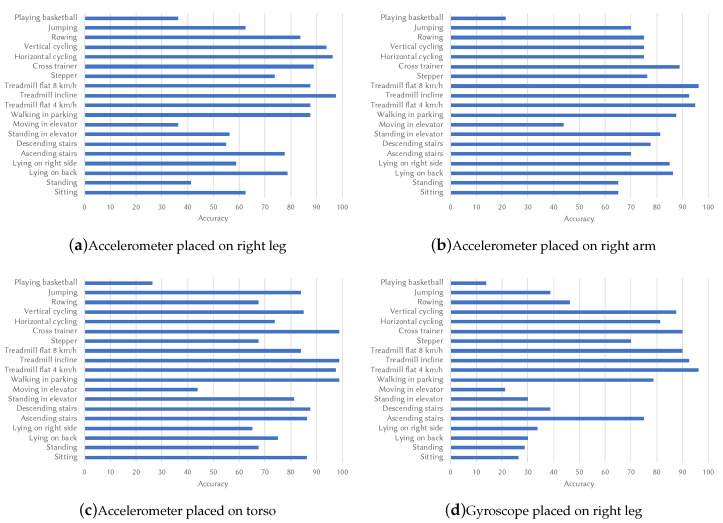
Single-sensor gait detection accuracy.

All 19 activities were tested in the same conditions, namely with 125 accelerometer values for each axis, 20 histogram bins, while using every sixth sample for testing and all the others as input into our solution. The accuracy results for placing an accelerometer on the right leg (i.e., keeping the smartphone in the trousers pocket) are presented in Figure 2a, showing that the activities with the highest accuracy values are cycling on a stationary bike (both vertical and horizontal), walking on an inclined treadmill and then on a flat treadmill, at both 4 and 8 km/h. This shows that the more repetitive activities have better scores and it can be assumed that the circular motion the leg does while cycling produces less noise. It can also be observed that all walking-related activities have high accuracy scores, which is encouraging for our gait detection-based authentication method.

With the sensor placed on the arm (as shown in Figure 2b), the accuracy results for the four different walking activities are among the highest as well, with values hovering around 90%. A decrease in the accuracy for cycling is observed, which may be caused by the placement of the arms on the handlebar of the stationary bike. Moreover, there is a great increase in the case of lying on the right side, which may be connected to the width of the torso, shoulders, and hips, which influences the angle of the body, depending on the position the candidates had. This improvement could also be explained by the different body structures of males and females.

For the third scenario, we tested with data collected when the sensor was attached to the torso, and the results are presented in Figure 2c. It can be observed that sitting accuracy increased the most, which may be explained by the different shapes of the torso, especially between genders. The walking activities scored the highest, maintaining the suitability of our solution for authentication using gait detection, as in the previous two cases. The stairs-related activities also show higher values, which may be a result of less noise propagating to the chest, which can greatly alter the results with a low frequency (this explanation may apply to jumping as well).

**Figure 3:**
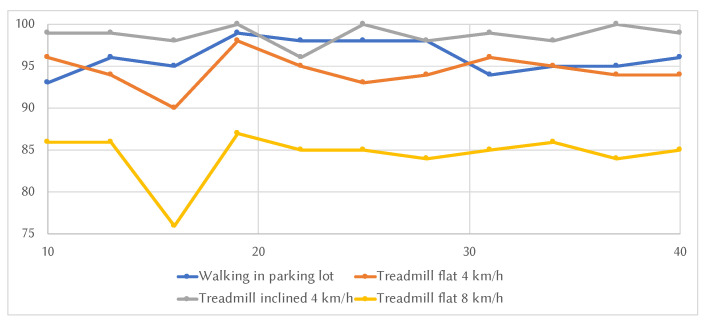
Variation of accuracy with different number of bins per histogram.

The next step in our evaluation was to vary the number of bins that compose the histograms and check how this parameter influences the accuracy. Since the most constant activities were the ones related to walking, only those were employed for this test, which was performed using only the accelerometer data, as in the previous scenarios. For this analysis, we varied the number of bins between 10 and 40, with a step of 3. With the sensor placed on the torso, all the activities peaked at 19 bins (as seen in Figure 3), further validating the default value chosen for the initial tests of 20 bins, which is about a sixth of the number of values per sample (125). However, this ratio may naturally vary with a higher number of sensor readings per sample (i.e., higher sampling rate or longer time intervals). In the case of the arm and leg placement of the sensor, the results were inconclusive since the activities oscillated more and, when one hit a maximum in accuracy, another one was at a minimum (although the results around the value of 20 were relatively high).

In order to choose the most suitable sensors, we applied the same methods as above for the data collected from the gyroscope. A high accuracy achieved using a second sensor would add another layer of security, since an attacker would have to replicate both the acceleration and the angular speed of the gait of the target to successfully authenticate. Furthermore, an additional sensor would also lead to an increase in the overall accuracy. After running tests across all 19 activities, the accuracy results were significantly lower. The chest and arm scenarios all had accuracy values well below 70%, except for walking on a flat treadmill at 8 km/h. However, on the data recorded from the leg (shown in Figure 2d), all the walking-related activities had results around 90%.

**Figure 4:**
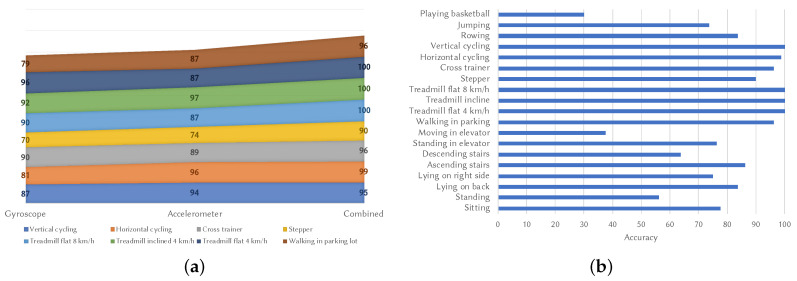
Gait recognition accuracy for various sensor combinations. (**a**) gait recognition accuracy variation for different sensor setups; (**b**) accelerometer and gyroscope placed on the right leg.

After applying the algorithm proposed in Section 3 on the data from both sensors and generating a histogram for each, the algorithm was modified to maximize the combined correlation between the acceleration histogram and the angular speed histogram, with the results shown in Figure 4a. In three out of the four walking activities, all samples were classified correctly, while, in the remaining one, the improvement was significant over the scenarios where a single sensor was employed.

Finally, Figure 4b shows the results for all the activities when two sensors are employed. It can be observed that some scenarios suffered decreases in accuracy, but the ones related to walking showed great improvements. The right leg was tested the most because the upper leg pocket is a common location for a mobile phone during walking and it is closer to a real-life scenario. Similar improvements were obtained for the torso and arm, especially for the walking activities. This validates the hypothesis that histogram similarity can be applied to the data obtained from the gyroscope as well. This means that this algorithm is a two-factor authentication model by itself. The fact that a completely correct classification can be obtained with a sampling rate as low as 25 Hz and an interval as short as 5 s demonstrates the feasibility of creating a continuous authentication algorithm and of combining it with classic methods.

However, a classification algorithm is unfit for the proposed application. Applying the information obtained from the classification tests, we extended the histogram comparison based algorithm to run with the enrolment set of a single user. To test this, we used a similar setup, by using 50 of the samples for each user as enrolment data and the rest as validation attempts. We initially compared the samples in each base set to find the minimum score between the trusted recordings. Then, each test set was checked against the base set of each user, by finding the average score a validation sample obtained and comparing it to the minimum previously computed. The minimum was multiplied with an error factor of 0.95 to take into consideration that the enrolment samples may be recorded in similar if not identical situations in the case of the data set. This means new samples, although valid, may score lower than existent data.

Based on the “Daily and Sports Activities Data Set”, we achieved the following result an overall accuracy of 94.69%. When comparing the samples of the same user, we calculated a false rejection rate of 6.25%. By simulating attacks on each user by the all the other users in the data set, we calculated a false acceptance rate of 5.19%.

Furthermore, we collected a custom data set by distributing the application to a number of acquaintances, to ensure proper communication with the tests subjects if needed. The sole criterion for participation was to have a smartphone running the Android operating system (version 5.0 or higher) at their disposal, which allowed the testing application to access both the accelerometer and gyroscope. In the end, gait information was gathered in the centralized data set from four females (ages 21, 23, 23, and 50) and six males (ages 24, 25, 25, 25, 50, and 52). None of the test subjects reported having medical conditions which may affect gait.

The users were instructed to register through Firebase Auth with a username, in order to label the data samples in the Firebase Firestore database for evaluation. Afterwards, when prepared to record the gait samples, they were instructed to start the recording from the application and place their mobile devices in the upper leg pocket of their clothing within a predefined interval (set to 5 s, to allow the proper placement of the phone before the actual recording begins). The subjects were asked to walk for a few minutes and were informed through local notifications each time a sample was completed.

To ensure the application will run without interruptions, the testing version was configured to keep the screen on and have a fixed orientation. We found this setting to be necessary because of the restrictions on background processing which are applied by some vendors to enhance battery life of their devices, in addition to the ones imposed by the operating system. These options cannot be changed from inside the app; however, they can be configured from the settings menu of the smartphone (Battery section). Since each vendor usually implements the settings menu differently, we wanted to avoid requesting the users to set up their devices.

Although the centralized database would facilitate the collection of data, we decided on this initial size of the data set based on the current environment. The ongoing pandemic required the imposition of outdoor travel restrictions. This leads to the collection of data being unsupervised, with no control over factors which may impact the information retrieved by the device such as: clothing, device positioning, travel trajectory, etc. In addition, non-essential trips were restricted so we reduced the number of samples to allow the recording to be completed during absolutely necessary travel, thus complying with current regulations.

This data set consists of 20 samples for each of the 10 subjects, recorded for 5 s with a sample rate of 100 Hz. As mentioned in this paper, motion sensors on smartphones can reach frequencies higher than 1000 Hz, but we encountered problems while managing samples of this frequency in the centralized database. For this test, the histograms were computed directly from the raw values retrieved from the motion sensors based on the formulas described in Section 3.2, with no further pre-processing in the algorithm. However, the sensor framework in Android provides two instances for the accelerometer and gyroscope, calibrated and uncalibrated. According to the Android documentation regarding motion sensors (https://developer.android.com/guide/topics/sensors/sensors_motion), the latter providing the values on the three axes without any bias or drift compensation, respectively. In this case, the calibrated instances of the sensors were used.

For this test, we split the samples in half for each user and reached an accuracy of 83% for the classification algorithm and 81.9% for the identification algorithm. Overall, we calculated a false rejection rate of 11% and a false acceptance rate of 18.77%. Following this test, high FAR values were observed for two of the users. Due to the unsupervised manner in which data collection was performed, we cannot identify the exact cause of these values or whether it is related to the algorithm itself or other external factors. We redid the test using a subset of eight candidates, achieving an FRR of 15% but with a FAR of 8.57%.

One factor which may impact the accuracy of gait-recognition is the positioning of the device. In testing, the Activity Recognition Client correctly identified walking in multiple situations: with the device in the upper leg pocket, with the device in hand swinging naturally and with the device in hand, static in relation to the body, as if being used. As shown, the algorithm is effective in various positions. However, processing different positions together may decrease the accuracy. To solve this issue, we experimented with clustering, by running the K-Means algorithm on the samples in the first data set. We considered the distance between two samples to be the difference between a perfect similarity score and the comparison score of the two samples. For evaluation, we checked the labels of the positions in each cluster. The “Daily and Sports Activities Data Set” contains five positions in each sample: torso, left arm, right arm, left leg, and right arm, so we targeted a number of five clusters for the K-Means algorithm. The results of the clustering algorithm. The results are shown in Table 1.

Analysing the positions in each cluster, it can be observed that if different body parts are grouped together in significant sample counts, it is usually the arms or the legs. Considering that, in the processing of the histogram, the orientation of the acceleration or rotation is not preserved; this is the expected behaviour. For the legs and arms, it can be assumed that, during data collection, the measuring device was placed in a similar position on the limb, which implies that some components of the acceleration and rotation vectors were symmetrical, which in turn translates into similar histograms.

By re-evaluating the first test and using the entire data set, with all positions and applying the clustering algorithm, we achieved the values in Table 2. The overall accuracy of the identification algorithm without clustering seems quite high. However, this is because almost all validation samples are rejected. However, with a false acceptance rate of 77.75%, the algorithm is rendered useless. By adding the K-Means algorithm into the equation, a slight decrease in accuracy is detected, but a great reduction of the false rejection rate can be observed. Although the false acceptance rate is doubled for five and eight clusters, the algorithm will be somewhat reliable. A positive trend can be observed in relation to the number of clusters. This may be explained by the increased similarity of samples inside the clusters. Taking into consideration that, in the application, the testing samples will be tested in batches with a majority vote, the accuracy may increase. An improvement to this algorithm would be to add a method of determining the optimal number of clusters. In this scenario, the number of positions was known; however, in a real-life scenario, the position of the device may be influenced not only directly by the user, but also by the clothing worn by the person.

## 5. Conclusions and Future Work

In this paper, we explored the possibilities offered by gait recognition in terms of mobile device security, through the implementation of an Android application that collects data from multiple sensors (such as accelerometer and gyroscope) and uses them as part of a multi-factor authentication mechanism, together with the default Android methods. The results obtained on a standardized data set show that the proposed algorithm implemented in the Android app is able to obtain good results in terms of gait recognition based on histogram similarity, with correct classification rates based solely on the accelerometer yielding values of 90%. Furthermore, by applying the same method to the gyroscope sensor and combining the result with the data from the accelerometer, an improved gait recognition algorithm was developed. The addition of a second sensor also means the addition of a new layer of security which further increases the difficulty of performing an impersonation attack. Mimicking the gait of a person is extremely hard even if the target can be closely observed by the attacker [15], so the probability of a successful attack is reduced even more since, in the proposed algorithm, the impersonator must replicate two sets of sensor data simultaneously. As a conclusion, we believe that gait authentication is a viable solution for user authentication on modern smartphones.

As future work, in order to make the implementation more secure, we want to attempt running the gait recognition algorithm in the Trusted Execution Environment offered by Android. It is a secure part of the device, which may have its own hardware and is also separated by software, running a secure operating system. Another improvement would be the addition of a cycle detection algorithm to synchronize the samples and compare this to a cycle detection based on the step counter available in Android. We attempted to use the virtual sensors provided by the Android operating system but discovered the step detection sensor may be inaccessible on most smartphones and it does not check for false positives. The step counter uses additional processing to discard false positives, but it reports events in batches with delays, which makes it inappropriate for time stamping the steps. Furthermore, we plan on testing our solution on larger data sets and improving its efficiency, in order to make it a more viable solution for Android devices. The data set gathered in the centralized database should be reconstructed in a more controlled environment and with a larger number of samples. These results will be used in comparisons between alternative solutions in the related literature, to better illustrate the behaviour and potential usage of the proposed solution in a real-life scenario.

## Figures and Tables

**Table 1:** Clustering on gait samples.

Activity	Cluster 1	Cluster 2	Cluster 3	Cluster 4	Cluster 5
Walking in parking lot	Torso: 58 Right arm: 2 Left arm: 1	Right arm: 58	Torso: 11 Left Arm: 59	Right leg: 32 Left leg: 19	Right leg: 28 Left leg: 41
Flat treadmill	Torso: 60 Left arm: 1	Right arm: 45 Left arm: 1	Left arm: 58	Right leg: 60 Left leg: 60	Right arm: 15
Inclined treadmill	Torso: 59 Right arm: 2 Left arm: 2	Torso: 1 Right arm: 9 Left arm: 52	Right leg: 36 Left leg: 23	Right arm: 1 Right leg: 24 Left leg: 37	Right arm: 48 Left arm: 6
Running	Torso: 60	Right arm: 12 Left arm: 12	Right leg: 60 Left leg: 60	Right arm: 1 Left arm: 48	Right arm: 47

**Table 2:** Clustering on gait samples.

	Accuracy	FAR	FRR
No clustering	80.56%	11.11%	77.75%
5 clusters	73.81%	27.46%	7.25%
8 clusters	78.62%	22.25%	5.25%
10 clusters	84.56%	16.96%	4.75%

## References

[1] Gafurov D., Helkala K., Sondrol T. (2006). Biometric Gait Authentication Using Accelerometer Sensor. JCP.

[2] Hayfron-Acquah J.B., Nixon M.S., Carter J.N. (2003). Automatic Gait Recognition by Symmetry Analysis. Pattern Recogn. Lett..

[3] Cunado D., Nixon M.S., Carter J.N. (2003). Automatic Extraction and Description of Human Gait Models for Recognition Purposes. Comput. Vis. Image Underst..

[4] Goethem T., Scheepers W., Preuveneers D., Joosen W. (2016). Accelerometer-Based Device Fingerprinting for Multi-factor Mobile Authentication. Proceedings of the 8th International Symposium on Engineering Secure Software and Systems, ESSoS 2016.

[5] Mantyjarvi J., Lindholm M., Vildjiounaite E., Makela S.M., Ailisto H. Identifying users of portable devices from gait pattern with accelerometers. Proceedings of the (ICASSP ’05) IEEE International Conference on Acoustics, Speech, and Signal Processing.

[6] Nickel C., Derawi M.O., Bours P., Busch C. Scenario test of accelerometer-based biometric gait recognition. Proceedings of the 2011 Third International Workshop on Security and Communication Networks (IWSCN).

[7] Sprager S., Juric M. (2015). Inertial sensor-based gait recognition: A review. Sensors.

[8] Abuhamad M., Abusnaina A., Nyang D., Mohaisen D. (2020). Sensor-based Continuous Authentication of Smartphones’ Users Using Behavioral Biometrics: A Survey. arXiv.

[9] Aviv A.J., Gibson K., Mossop E., Blaze M., Smith J.M. (2010). Smudge Attacks on Smartphone Touch Screens. Proceedings of the 4th USENIX Conference on Offensive Technologies (WOOT’10).

[10] Galbally-Herrero J., Fierrez-Aguilar J., Rodriguez-Gonzalez J., Alonso-Fernandez F., Ortega-Garcia J., Tapiador M. On the vulnerability of fingerprint verification systems to fake fingerprints attacks. Proceedings of the 40th Annual 2006 International Carnahan Conference on Security Technology.

[11] Cao K., Jain A.K. (2016). Hacking Mobile Phones Using 2D Printed Fingerprints.

[12] Gupta S., Buriro A., Crispo B. (2018). Demystifying authentication concepts in smartphones: Ways and types to secure access. Mob. Inf. Syst..

[13] Swain M.J., Ballard D.H. (1991). Color indexing. Int. J. Comput. Vis..

[14] Maji S., Berg A.C., Malik J. Classification using intersection kernel support vector machines is efficient. Proceedings of the 2008 IEEE Conference on Computer Vision and Pattern Recognition.

[15] Gafurov D., Snekkenes E., Buvarp T.E. (2006). Robustness of biometric gait authentication against impersonation attack. Proceedings of the OTM Confederated International Conferences “On the Move to Meaningful Internet Systems”.

